# Factors contributing to 25-year-long retention of South Australian general practitioners in rural practice: a cross-sectional survey

**DOI:** 10.1186/s12913-026-14522-1

**Published:** 2026-04-15

**Authors:** Richard Watts, Drishti Gupta, Amy Watts, Tharun Kathiravan, Prathicksha Venkatesan

**Affiliations:** 1 Department of Anaesthesia, The Queen Elizabeth Hospital, 28 Woodville Rd, Woodville South, Woodville, SA 5011 Australia; 2 Discipline of Rural Health, Flinders University, Sturt Rd, Bedford Park, SA 5042 Australia; 3 School of Medicine, University of Adelaide, 4 North Tce, Adelaide, SA 5000 Australia; 4 RACGP Rural Registrar, Angaston Medical Centre, 46 Murray St, Angaston, SA 5353 Australia; 5 The Queen Elizabeth Hospital, 28 Woodville Rd, Woodville South, SA 5011 Australia

**Keywords:** Rural health, Health personnel, Community healthcare, Professional practice

## Abstract

**Introduction/Background:**

The difficulty in retaining general practitioners (GPs) in rural and remote parts of Australia is well recognised. Fewer GPs ultimately means patients are more likely to wait longer for GP services, and experience poorer health outcomes as compared to their urban counterparts.

**Objectives:**

To investigate the perceived factors that influenced the long-term retention of GPs in rural South Australia (SA).

**Method/Design:**

Cross-sectional survey with questions assessing three main domains of personal, professional and social factors.

**Setting and participants:**

GPs who have served twenty-five years in rural areas of SA are presented with a Long Service Award. These GPs were invited to participate over email with the questionnaire sent using Survey Monkey.

**Main outcome measures:**

Retention factors were assessed across the domains: personal factors related to demographics, background, training, type of practice; professional factors related to scope of practice and finance, and social factors related to lifestyle and extracurricular activities.

**Results:**

Members of the Rural Doctors Workforce Agency of South Australia who had served 25 years in rural areas of SA (*n* = 142) were invited to participate and 60 responded. Three-quarters had rural experience as a medical student and had over 30 years’ experience in rural practice. Involvement in hospital care, emergency medicine/on call, teaching, indemnity support and on-call allowance were the top professional factors perceived as influencing retention. A sense of community, flexible shared after-hours work, regular holidays, friendship groups, community respect, sports, participating in rural students’ elective placement and volunteering were the factors linked to social aspects.

**Conclusion:**

The current study sought to identify what factors GPs perceived influenced their decision to practice in rural or remote areas. This is crucial for understanding the key factors the next generation of rural GPs. Promoting rural medical student experience, arrangements with hospital and procedure-related practice, financial support, a shared workload, and a sense of community may encourage GPs to remain and serve in rural areas. Understanding the factors derived from a cohort of long-serving rural GPs is advantageous for policymakers to implement targeted retention strategies tailored to the specific factors.

**Supplementary Information:**

The online version contains supplementary material available at 10.1186/s12913-026-14522-1.

## Introduction

The difficulty in retaining general practitioners (GPs) in rural and remote areas in Australia are well recognised [1, 2]. In remote areas, the number of GP services per capita is about 60% of that available in a major city [3]. This impacts primary health care delivery as patients are likelier to wait longer to access GP services, and concurrently these populations experience poor health outcomes as compared to their urban counterparts [4, 5]. Retention has been extensively studied with evidence suggesting that medical students with a rural background and/or positive rural experience during training are more likely to commence and remain in rural practice [1, 6, 7]. Doctors who undergo training in the Remote Vocational Training Scheme are likely to stay in the same geographical location for a longer period of time. This was evident in a recently published Australian study that assessed 506 participants who were enrolled in the RVTS between 2000–2023 [8]. There is further evidence that highlights longer duration of training in one specific rural region (> 12 weeks) is likely to increase likelihood of doctors working in the same region, and can be a key strategy to building workforce capacity [9]. Medical students who are likely to have rural immersion for more than a year are likely to return to the same region to practice after graduation [10]. On a similar note, rural training as a medical student was noted to be a significant factor for long-term retention in any rural region [7]. Other influencing factors include demographic and personal attributes, nature of work including procedural work, hospital work, availability of academic and professional support, social, educational, and recreational opportunities, financial incentives such as primary income source, and work-life balance considerations [11–16]. Practice ownership was associated with a 70% higher retention than average, whilst ability to practice in hospitals retained GPs to approximately 18% higher than average [16]. Russell et al. also highlighted the importance of bundling retention strategies and attempting to address a combination of modifiable retention factors [15].

The literature is predominantly based on cross-sectional surveys with few using longitudinal approaches to identify factors vital for retention [2, 14]. Very few of the studies evaluate long-term retention, and in many studies length of service is not documented [2, 14]. One longitudinal study, however, used survival analyses to guide retention strategies and found that Australian trained family physicians with public hospital admitting rights were likely to remain in service for 11 years in small closely settled coastal locations [17]. They also found strong associations between geographical location and population size in retention [17]. Russell et al. conducted a study and tentatively developed retention benchmarks for length of service, suggesting a period of three years in rural locations and two years in remote regions [18]. McGrail and Humphreys echoed the above findings, concluding that GPs who worked in small communities and in rural locations for less than 3 years were at highest risk of leaving rural practice [19]. With the wide range of retention factors proposed, a longitudinal prospective study or a framework to compare a suite or ‘bundle’ of factors to standard conditions would be ideal but impractical. A ‘surrogate’ alternative to this would be to conduct a ‘retrospective’ cross-sectional study of GPs who had already provided long service to a rural community to investigate what retention factors were deemed important. The objective of this study was to explore the perceived factors that influenced the 25-year-long retention of GPs in rural South Australia (SA).

## Methods

The study was approved by the local ethics committee (ref no: 14167). Informed consent was obtained from all participants and this was implied through completion of the questionnaire and in accordance with the National Health and Medical Research Council (NHMRC) statement of ethics. An early systematic review of effective retention incentives for Australian rural and remote healthcare workers in 2010 analysed six program evaluation articles, eight review articles and one literature report. The review found a six-component framework for retention, comprising of staffing, infrastructure, remuneration, workplace organisation, professional environment and social, family and community support [20]. It concluded there was scant evidence to suggest the use of one specific strategy, and that multiple retention strategies needed to be utilised, targeting the six main retention factors. A subsequent Australian systematic review published in 2017 [15] has also supported the conclusion of multiple factors contributing to retention, with the factors overlapping with the aforementioned study seven years prior [20]. Developing a strategy specifically aimed towards key retention factors was also described.

As such, a questionnaire was created to assess the range of retention factors under 3 main domains: (a) personal factors related to demographics, background, training, type of practice and location; (b) professional factors related to scope of practice and finance, and (c) social factors related to lifestyle and extracurricular activities. Each personal factor was assessed as a binominal response. For example, whether they received rural exposure as a medical student was assessed using “Yes/No”, whereas their current status was determined using “Practicing/Retired”. The extent to how important each professional and social factor was for long-term retention was assessed, with the following question posed: “How important was (insert professional/social factor) in retaining you to work in rural South Australia?” Specific definitions for each factor were not provided, but rather left to the participants to self-define. For example, we asked about “Obstetrics” but did not expand the scope of importance as “to have more/less exposure” or “be protected from this service”.

The responses for the professional and social factors were ranked as: (a) very important; (b) somewhat important; and (c) not at all important.

An example of a survey question shown to respondents is illustrated below:

Medical indemnity support:

- Very important
- Somewhat important
- Not at all important

The survey was pilot tested across five GPs to assess comprehension and overall flow. No amendments were made after pilot testing and it was decided to exclude the results in the final data set. The Rural Doctors Workforce Agency of South Australia presents GPs who have served twenty-five years in rural and remote areas of SA with a Long Service Award. These members were invited by email by the RDWA team, and the questionnaire was sent using Survey Monkey. A reminder invitation was sent after two weeks, and responses were analysed using Microsoft Excel 2018. Data are expressed as percentage of responses to each questionnaire. No formal statistical analysis was applied and the results were analysed and presented in a descriptive format.

## Results

Invitations were sent to 142 potential participants. Sixty participants completed the survey between 28th October 2022 and 28th November 2022, yielding a response rate of 42%. Missing responses for a specific factor were not included in expressing the results.

### Personal factors

Over half of them were above 65 years (32/60, 53%) and one-third had rural background (20/60, 33%). Three-quarters had rural experience as a medical student (45/60, 75%) and accumulated over 30 years in rural practice (46/60, 76%), and 81% (48/60) were male and current practitioners. The majority were involved in group practice (52/60, 86%) (Table 1).

To simplify the analysis, only the responses under *‘very important’* were used to establish the ranking of retention factors in terms of significance.

**Table 1:** Demographic characteristics and personal factors associated with retention of general practitioners, *n* = 60

	*N* (%)
*Demographic characteristics*	
Age (years)	
50–55	1 (2)
55–60	12 (20)
60–65	15 (25)
65–70	19 (32)
>70	13 (22)
Male	48 (80)
*Personal factors*	
Rural background	20 (34)
Overseas trained	26 (43)
Spouse with rural background	19 (32)
Rural experience as medical student	45 (75)
Total years in rural practice*	
25–30	13 (22)
>30	46 (78)
Years in current/final practice	
1–5	3 (5)
5–10	3 (5)
>10	51 (89)
Currently practicing	48 (81)
Type of practice most spent	
Solo	7 (12)
Group	52 (87)
Locum	3 (5)
Geographic location most spent	
Coastal	22 (37)
Inland	39 (65)

N = number of individuals

* Data represents results from 59 respondents

### Professional factors

**Fig. 1:**
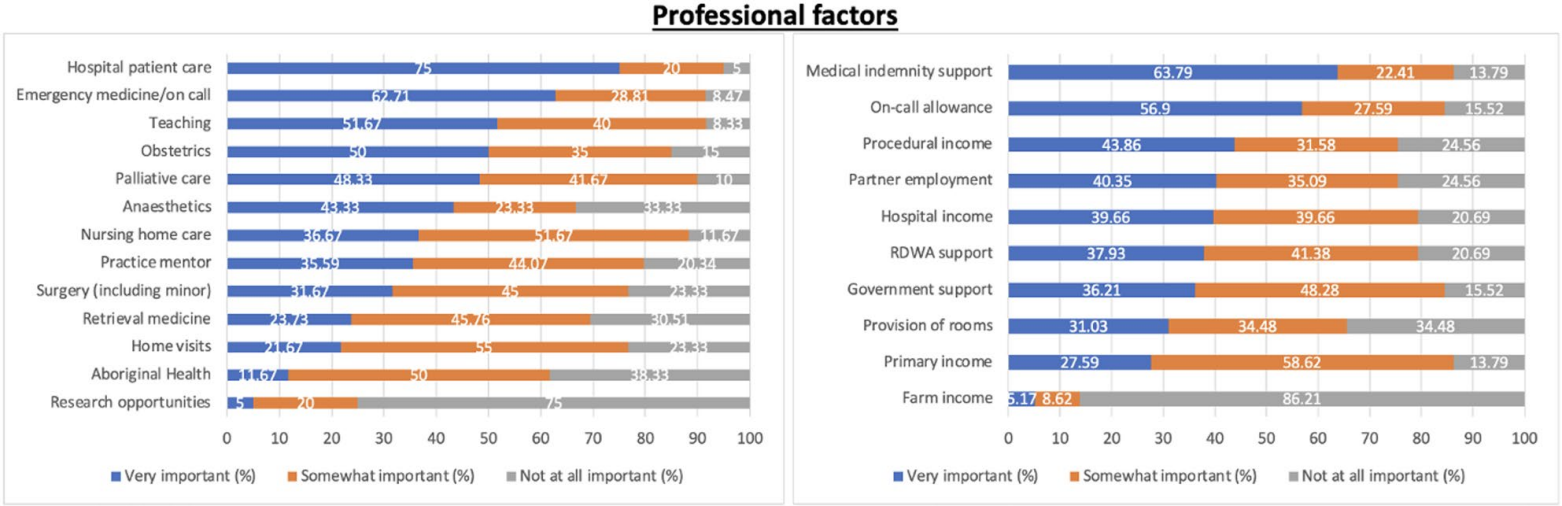
Professional factors encompassing scope of practice and financial attributes as determinants of retention in rural practice

Of the professional factors related to scope of practice, involvement in hospital patient care, emergency medicine/on call, and teaching students and registrars were the top three factors. Home visits, nursing home care and Aboriginal health were the three factors most commonly selected as “somewhat important”. Research participation, Aboriginal health and anaesthetics were the least prioritised as “not at all important” (Fig. 1). Among the professional factors linked to financial aspects, medical indemnity support and on-call allowance were rated as topmost factors, followed by procedural income. Primary income, government support and RDWA support were listed most often as “somewhat important”. Factors selected as “not at all important” in order of popularity include farm income and provision of rooms.

### Social factors

**Fig. 2:**
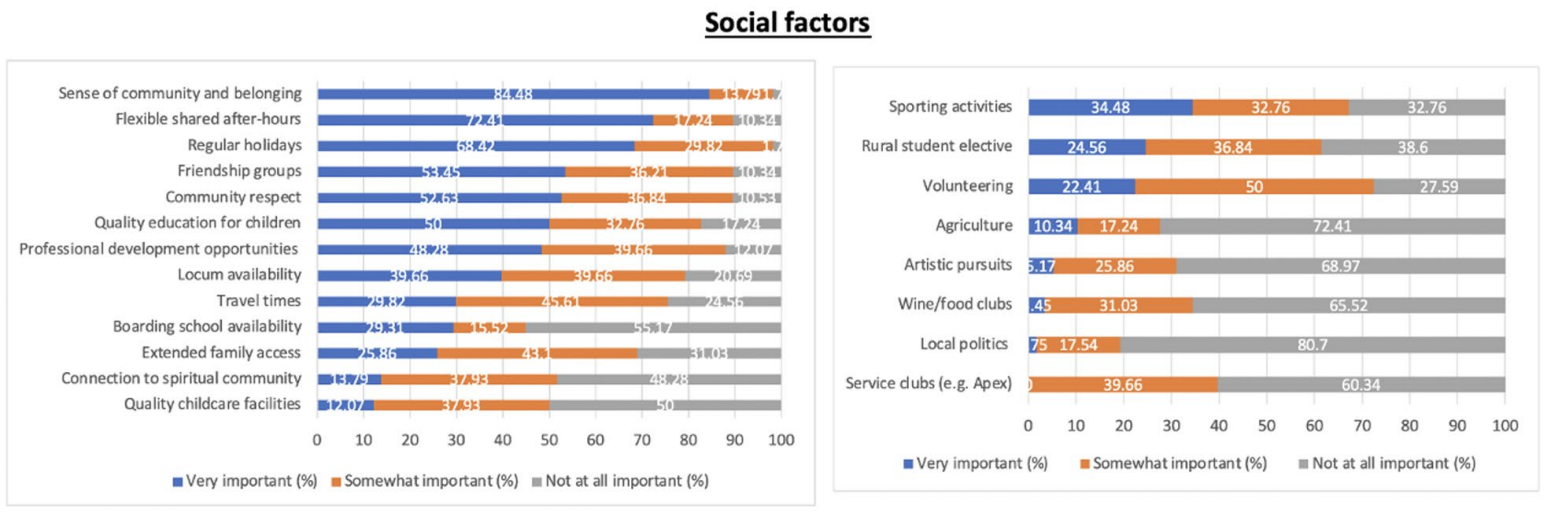
Social factors encompassing lifestyle and extracurricular activities as determinants of retention in rural practice

Of the social factors related to lifestyle, a sense of community, flexible shared after-hours work and regular holidays were rated by the participants as important factors (Fig. 2). Travel times and extended family access were considered as most commonly “somewhat important”. Boarding school availability, quality childcare facilities and connection to spiritual community were selected as being least important amongst respondents. Among the social factors related to extracurricular activities, sports, participating in rural students’ elective placement and volunteering were rated as the top three factors. Volunteering, service clubs and rural student electives were the three factors commonly selected as “somewhat important”. The three factors considered as “not at all important” were local politics, agriculture and artistic pursuits.

## Discussion

The challenges with long-term retention of GPs in rural/remote Australia and the subsequent impacts on health care are well known. This is evidenced as per a study on GPs training in the Remote Vocational Training Scheme (RVTS) finding that over the long-term, only 18.2% were retained in the same community [8]. It is crucial to firstly identify the key factors that are essential for retaining rural GPs. This will provide a foundation for developing specific strategies to address these factors. This information will be valuable for policymakers to implement strategies for retaining the next generation of rural GPs.

Our study found that university rural experiences, hospital engagement, emergency/on call arrangements, teaching, indemnity support, on-call allowances, procedural income, sense of community, flexible shared after-hour work commitments and regular holidays were key factors that were perceived to influence retention of long-serving GPs in rural SA.

A rural background, rural clinical school experience, and a partner of rural background were found to be strong predictors for rural medical practice in many Australian studies [21–24] and a systematic review [1]. In our study, two-thirds of the participants admitted to have a non-rural upbringing. However, this was considered as a background/demographic characteristic and hence, it does not imply a causal relationship as a factor influencing retention. Additionally, our study found that 75% had rural clinical experience as a student, and approximately one-third had a spouse/partner with a rural upbringing, highlighting that previous rural exposure is an important factor in the retention of practitioners in rural practice. The mean age of rural GPs in Australia in 2014 was 50 years, which is much lower than in our study [25]. However, this discrepancy is likely because we have only included GPs who have served at least 25 years. In our cohort, only 20% of respondents identified as female. This was lower than the percentage reported by Australian rural medical employment agency in 2018 who found that 41% of rural GPs identified as female [26]. Recruiting students from rural areas [1] and providing ample training in these areas [14] with rural GP mentors [27] has positively influenced retention. Makate and authors have also determined that a prior rural background is likely to be positively correlated with future rural practice [28]. Extended experience as a medical student in rural settings has been found to significantly improve likelihood of long-term rural retention [29]. A recent 2024 Australian study found establishment of connectedness – encompassing personal, professional and geographic – as a significant factor contributing to rural retention [30]). The study suggested that prior rural experience played a key role in fostering this connectedness. Postgraduate training options in rural and remote areas [15], combined with financial, professional and social factors also remain the cornerstones of recruitment and retention [15, 20]. Our survey results reiterate this view.

An Australian survey across 1400 rural GPs revealed that on-call arrangements, professional support and procedural engagement were important retention factors [31]. Our results mirror these and reiterate that absence of adequate on-call arrangements and flexible shared after-hours workload may impose a relentless burden. Therefore, this may negatively impact the wellbeing of GPs and their families, making the rural locations less attractive [31]. To prevent GPs leaving rural practice prematurely, location-specific strategies should be implemented to ensure adequate support for on-call and after-hours work [31]. Uniquely, our respondents rated teaching as one of the top-three factors related to scope of practice. Data on this aspect is scant; intuitively, it implies that long-serving GPs had a sense of commitment to disseminate their clinical acumen to the future workforce. Paynter et al. conducted a study on sixty-six rurally-inclined Australian medical students, attempting to establish the preferences of the next generation of rural doctors. They found career diversity and lifestyle/community to be the two strongest factors. Whilst our study did not explore career diversity directly, sense of community was also rated as the most important social factor [32].

Various possible ethical issues were considered while designing the study. Involvement in Aboriginal health was given less importance by our participants which may relate to the age of the surveyed cohort. It has to be acknowledged, however, that the opportunity to get involved in Aboriginal health is available in selected pockets of rural Australia. Our findings in this regard may not reflect the perceptions of contemporary practitioners. In the process of constructing and completing this research, all possible ethical issues were considered and reviewed by the ethics committee, thus negating any conflicts of interest.

Indemnity support and income were highly rated financial factors in our study as previously shown [16]. GPs may choose rural practice to participate in a variety of procedural skills, for which their associated procedural income was ranked highly as a factor in our study. Assessments using length of stay data from a study analysing two Australian GP workforce datasets from the 2008 Australian GP workforce showed that undertaking hospital work was associated with a 18% higher retention rate [16]. This was reflected in our survey where continuity of care as hospital work, obstetrics and palliative care were ranked highly. A one-year diploma of rural generalist anaesthesia programme has been recently launched as a collaboration across the Australian and New Zealand College of Anaesthetists, the Australian College of Rural and Remote Medicine and the Royal Australian College of General Practitioners. This could potentially encourage future GPs by providing additional specialised training in an area of interest. This training will encourage the next generation of rural GPs. The Rural Procedural Grants Program for skills maintenance and upskilling is also a welcomed initiative [33]. If there are no hospitals in their primary area of practice, GPs can undertake procedural work in available nearby towns.

A 2019 Australian study found that community integration and work-life balance encourages rural attraction and retention of GP workforce [14]. Here, our results affirmed that sporting activities and volunteering had a positive impact in retention possibly enhancing community integration and reducing burnout.

It must be noted, however, that our findings are from participants who practiced in a distinct era. The contemporary workplace is demographically different with a higher proportion of female doctors, doctors with different career stage pressures who are working under different general practice models, and an era where the political, social and economic issues around rural medicine are different. O’Sullivan et al. assessed in their study the effect of formal national training policies that fund rural training experiences for medical students. While rural training has increased, there are fewer general practitioners with a higher proportion of females. This limits the impact of the rural training policy and highlights that modern cohorts are different to previous generations [7].

Policy makers and employers must focus on the social, professional and financial aspects together when deciding on and implementing retention strategies. Specific policies could include increasing the duration of mandatory rural placements during medical school, increasing exposure to hospital patient care including work in the emergency department, improving financial incentives by increasing on-call allowance, and engagement in community activities to foster a sense of social belonging.

Our study had several strengths. The study cohort was homogeneous with well-defined duration of rural service in SA, and our findings may be generalisable to their interstate peers. Multiple domains with the perceived ability to influence retention were explored. The vast majority (81%) of respondents were still working rurally at the time of the study. Yet, recall bias is highly likely as retention factors can potentially change over lengthy career spans. However, there are multiple limitations. The quality of survey is a key limitation. A thorough literature search was conducted to identify the factors listed in the survey. Yet it is possible that other vital factors may have been overlooked. It is likely that the relative importance of each variable would have changed across the respondents’ career spanning over 25 years. This was not explored in our survey and this is a further limitation. Another limitation was that our questionnaire did not address specific details about each professional and social factor. Although the intention of the survey questionnaire was to ascertain participants’ responses in a positive direction (e.g. towards the attractiveness of emergency/on call), the questionnaire battery did not explicitly mention this. Therefore, we cannot rule out the respondents interpreting this in a negative direction. Our study primarily surveyed relatively senior and male GPs whose careers largely pre-date contemporary rural clinical school, rural generalist, and training policy settings; thus, findings should be interpreted within this historical context and may not be applicable to graduates of the current generation and younger GPs. A younger cohort might value a different range of factors, although supporting evidence is limited and this was not explored in our study. Younger GPs have a tendency to try rural practice early, and then move out for the betterment of their children’s education [21]. Although multiple domains were explored, unidentified confounders may have influenced our findings. Additionally, certain retention factors were not clearly defined (e.g. the amount of teaching, number of holidays) and left at the discretion of participants. Stricter definitions may have yielded different results. Since the variables explored in this survey were self-defining in nature, the results could only illustrate perceived factors and not actual factors. Our survey did not assess the proportion of participants who were business owners. The responsibilities associated with managing a GP practice may have influenced results. If participants have never practiced in a specific area, for instance obstetrics or Aboriginal health, they are likely to perceive it as less important for retention. This represents a confirmation bias of our study. A further limitation of our study is the relatively small sample size of 60 participants – a national or international study would have provided a larger sample size and allowed us to apply robust statistical methods such as multivariable logistic regression to analyse the weight of individual factors. Potential response bias resulting from a low response rate of 42% is another limitation. Additionally, our survey was not validated beyond basic pilot testing. Sampling bias is possible, however we are unable to confirm or deny this as we did not compare the characteristics of respondents with non-respondents. The high percentage of male participants could have skewed results and since there is an increasing proportion of female doctors in the contemporary workplace, the most important retention factors may vary. The rurality of GPs was not assessed, with location of practice potentially changing importance of retention factors.

## Conclusion

Our survey highlighted factors that participants perceived to have influenced their 25-year-long retention of GPs in rural SA. Rural experience as a medical student, engagement in patient management in hospital, emergency medicine/on call arrangements, teaching, supportive framework for medical indemnity, on-call allowances and procedural income were perceived as vital factors that may have encouraged GPs to remain rurally and serve for a long period of time. A sense and engagement in community and flexible shared working hours were further important factors. The proportion of graduates entering GP training is declining and recently it has become a challenge to attract and retain younger doctors in the program. This is likely to have a flow-on effect to the supply of rural workforce. Policymakers should consider this factor in implementing strategies to enhance retention in rural communities.

## Supplementary Information

Below is the link to the electronic supplementary material.

Supplementary Material 1

## Data Availability

All data generated or analysed during this study are included in this published article and its supplementary information files.

## Abbreviations

GP: General practitioner
SA: South Australia
