# Supplementary material for: Factors contributing to 25-year-long retention of South Australian general practitioners in rural practice: a cross-sectional survey
Source: BMC Health Serv Res. 2026 Apr 15;26:751. doi: 10.1186/s12913-026-14522-1 (PMC13202836; doi:10.1186/s12913-026-14522-1)
Supplement: Supplementary file 1 — Supplementary Material 1 [file 12913_2026_14522_MOESM1_ESM.docx]

**Survey of long-serving rural and remote GPs**

**Demographics**

100 Age

1. 45- 50 years
2. 50-55 years
3. 55-60 years
4. 60-65 years
5. 65-70 years
6. > 70 years

101 Gender

1. Male
2. Female
3. Other

102 Rural background

1. Yes
2. No

103 Spouse/partner with rural background

1. Yes
2. No

104 Overseas trained

1. Yes
2. No

105 Rural experience as a medical student

1. Yes
2. No

106 Total years in rural practice (free response)

1. 20-25 years
2. 25-30 years
3. > 30 years

107 Years in current/final practice (free response)

1. 1-5 years
2. 5-10 years
3. > 10 years

108 Current status

1. Practicing
2. Retired

**Type of practice in which you spent most of your career**

109 Solo

1. Yes
2. No

110 Group practice

1. Yes
2. No

111 Locum

1. Yes
2. No

**Geographic Location**

112 Coastal

1. Yes
2. No

113 Inland

1. Yes
2. No

**Location**

114 Postcode (free response)

**In addition, how important were each of the following factors in your decision to remain in rural practice?**

**Domain 1: Scope of Practice**

Importance to Retention

201 Obstetrics

1. Very Important
2. Somewhat Important
3. Not at all Important

202 Anaesthetics

1. Very Important
2. Somewhat Important
3. Not at all Important

203 Emergency medicine/on call

1. Very Important
2. Somewhat Important
3. Not at all Important

204 Surgery (including minor)

1. Very Important
2. Somewhat Important
3. Not at all Important

205 Retrieval medicine

1. Very Important
2. Somewhat Important
3. Not at all Important

206 Hospital patient care (continuity)

1. Very Important
2. Somewhat Important
3. Not at all Important

207 Aboriginal health

1. Very Important
2. Somewhat Important
3. Not at all Important

208 Palliative care

1. Very Important
2. Somewhat Important
3. Not at all Important

209 Nursing home care

1. Very Important
2. Somewhat Important
3. Not at all Important

210 Ease in providing home visits

1. Very Important
2. Somewhat Important
3. Not at all Important

211 Research opportunities

1. Very Important
2. Somewhat Important
3. Not at all Important

212 Teaching (students/registrars)

1. Very Important
2. Somewhat Important
3. Not at all Important

213 Practice mentor

1. Very Important
2. Somewhat Important
3. Not at all Important

214 How did the importance of these factors change over the course of your practice? (Free response)

215 Are there any factors which were at play? (free response)

216 What would you change about your practice to make it ideal? (free response)

**Domain 2: Lifestyle**

Importance to Retention

301 Short travel times

1. Very Important
2. Somewhat Important
3. Not at all Important

302 Sense of community and belonging

1. Very Important
2. Somewhat Important
3. Not at all Important

303 Connection to spiritual community

1. Very Important
2. Somewhat Important
3. Not at all Important

304 Community respect

1. Very Important
2. Somewhat Important
3. Not at all Important

305 Extended family access

1. Very Important
2. Somewhat Important
3. Not at all Important

306 Friendship groups

1. Very Important
2. Somewhat Important
3. Not at all Important

307 Quality education for children

1. Very Important
2. Somewhat Important
3. Not at all Important

308 Opportunities for professional development

1. Very Important
2. Somewhat Important
3. Not at all Important

309 Availability of boarding schools

1. Very Important
2. Somewhat Important
3. Not at all Important

310 Regular holidays

1. Very Important
2. Somewhat Important
3. Not at all Important

311 Quality childcare facilities

1. Very Important
2. Somewhat Important
3. Not at all Important

312 Locum availability

1. Very Important
2. Somewhat Important
3. Not at all Important

313 Flexible shared after-hours

1. Very Important
2. Somewhat Important
3. Not at all Important

314 Do you feel that you have a good work-life balance? (free response)

315 Are you just practicing until you can retire? (free response)

**Domain 3: Financial**

Importance to Retention

401 Income

1. Very Important
2. Somewhat Important
3. Not at all Important

402 Gov. support (e.g. P/P, rural incentives etc.)

1. Very Important
2. Somewhat Important
3. Not at all Important

403 RDWA support

1. Very Important
2. Somewhat Important
3. Not at all Important

404 Hospital income (fee for service)

1. Very Important
2. Somewhat Important
3. Not at all Important

405 Procedural income

1. Very Important
2. Somewhat Important
3. Not at all Important

406 Provision of rooms

1. Very Important
2. Somewhat Important
3. Not at all Important

407 Partner (spouse) employment opportunities

1. Very Important
2. Somewhat Important
3. Not at all Important

408 On-call allowance

1. Very Important
2. Somewhat Important
3. Not at all Important

409 Medical indemnity support

1. Very Important
2. Somewhat Important
3. Not at all Important

410 Farm income

1. Very Important
2. Somewhat Important
3. Not at all Important

411 Do you have a long-term plan in place for work/leisure/retirement? (free response)

412 Briefly, what is it? (free response)

**Domain 4: Extracurricular activities**

Importance to Retention

501 Sporting activities (sailing, cricket, tennis etc.)

1. Very Important
2. Somewhat Important
3. Not at all Important

502 Agriculture (farming, grapes)

1. Very Important
2. Somewhat Important
3. Not at all Important

503 Service clubs (e.g. Apex)

1. Very Important
2. Somewhat Important
3. Not at all Important

504 Wine/Food clubs

1. Very Important
2. Somewhat Important
3. Not at all Important

505 Artistic pursuits (acting, music, painting)

1. Very Important
2. Somewhat Important
3. Not at all Important

506 Local politics

1. Very Important
2. Somewhat Important
3. Not at all Important

507 Volunteering

1. Very Important
2. Somewhat Important
3. Not at all Important

508 Rural student elective

1. Very Important
2. Somewhat Important
3. Not at all Important

509 Do you leave the town to enjoy yourself? (free response)

510 Do you feel you have to leave for anonymity? (free response)
